# Vulvar neuroendocrine carcinoma that is independent of merkel cell polyomavirus and human papillomavirus suggests endometrial cancer recurrence: a case report

**DOI:** 10.1186/s12902-022-00987-8

**Published:** 2022-03-29

**Authors:** Tomoko Hirakawa, Mitsutake Yano, Haruto Nishida, Shimpei Sato, Kaei Nasu

**Affiliations:** 1 Department of Obstetrics and Gynecology, Faculty of Medicine, Oita University, 1-1 Idaigaoka, Hasama-machi, Yufu-shi, Oita, 879-5593 Japan; 2 Department of Diagnostic Pathology, Faculty of Medicine, Oita University, 1-1 Idaigaoka, Hasama-machi, Yufu-shi, Oita, 879-5593 Japan; 3 Division of Obstetrics and Gynecology, Support System for Community Medicine, Faculty of Medicine, Oita University, 1-1 Idaigaoka, Hasama-machi, Yufu-shi, Oita, 879-5593 Japan

**Keywords:** Neuroendocrine carcinoma, Vulva, Endometrial carcinoma, Metastasis, Merkel cell carcinoma, Human papillomavirus, Case report

## Abstract

**Background:**

Vulvar neuroendocrine carcinomas with small cell morphology need an appropriate differential diagnosis with respect to primary Merkel cell carcinomas, primary small cell neuroendocrine carcinomas, and secondary/metastatic carcinomas. Herein, we report a woman with a history of endometrial carcinoma led to neuroendocrine vulvar carcinoma.

**Case presentation:**

An 82-y-old woman with right vulvar swelling was transferred to our hospital. Computed tomography scan showed a 75 mm irregular mass in her right vulva. Three years ago, she had been diagnosed with endometrial endometrioid carcinoma stage IA and had undergone surgery. Vulvar biopsy revealed neuroendocrine carcinomas with small cell morphology. Immunohistochemical staining showed that the vulvar tumor was positive for CD56 and chromogranin A, but negative for Merkel cell polyomavirus and cytokeratin 20. Incidentally, her endometrial carcinoma was also positive for CD56 and chromogranin A. Human papillomavirus DNA typing analysis of vulvar tumor was negative. Hence, the vulvar tumor seemed to be a recurrence of the endometrial cancer rather than a primary vulvar neuroendocrine carcinoma. The patient died of the disease within a month.

**Conclusion:**

We report a case of vulvar neuroendocrine carcinoma that is independent of Merkel cell polyomavirus and human papillomavirus, thereby suggesting a recurrence of endometrial cancer. Immunohistochemical and virological analyses helped in the differential diagnosis of the neuroendocrine carcinoma.

## Background

High-grade neuroendocrine carcinomas (HGNECs) of the vulva are extremely rare, but when they occur, they usually present as aggressive neoplasms [1]. These tumors can be further classified into Merkel cell carcinoma, small cell neuroendocrine carcinoma (SCNEC), and large cell neuroendocrine carcinoma according to morphological, immunohistochemical, and virological characteristics. Previously, most of the vulvar HGNECs were considered to be Merkel cell carcinomas [1]. However, Chen et al. [1] performed detailed immunohistochemical and virological analyses of 12 HGNECs with pure small cell morphology and reported that out of those 12 cases, 6 (50%) were SCNECs and 6 (50%) were Merkel cell carcinomas. The primary vulvar Merkel cell carcinoma is immunohistochemically positive for Merkel cell polyomavirus (MCPyV) and cytokeratin (CK) 20 with a paranuclear dot-like and/or ring-like reactivity, while the primary vulvar SCNEC is associated with high-risk human papillomavirus (HR-HPV) infection [1].

The HGNECs can also occur in the uterine endometrium, and they often coexist with endometrioid carcinomas [2–4]. In fact, Ono et al. [3] had reported that a case of endometrial cancer, which had been initially diagnosed as endometrioid carcinoma, exhibited HGNECs in the lungs during recurrence/metastasis. Similarly, D'Antonio et al. [4] had reported a case of endometrial cancer with coexisting endometrioid carcinoma and SCNEC where only the SCNEC had metastasized to the lungs and lymph nodes. Due to under-recognition of the HGNEC components, most endometrial HGNECs (89%) represent a diagnostic disagreement, and they have been interpreted as endometrioid carcinoma or other histological types [2].

Herein, we report the case of a woman with a history of endometrial endometrioid carcinoma that led to vulvar HGNEC. Immunohistochemical and virological analyses suggested recurrence of endometrial cancer rather than development of a primary vulvar HGNEC.

## Case presentation

### Clinical history

**Fig. 1:**
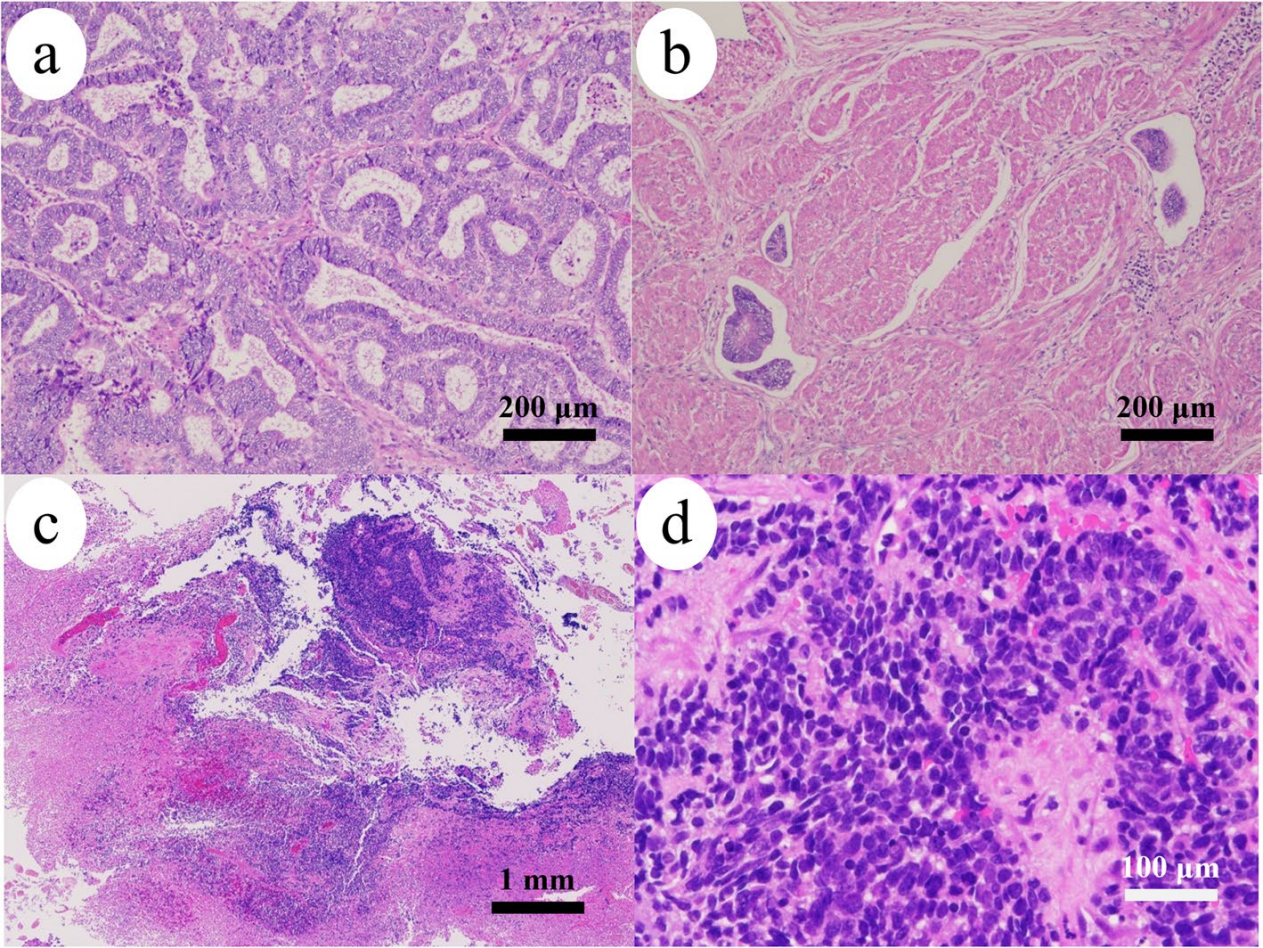
Histological findings (Hematoxylin and Eosin staining): **a** (× 10) – Hysterectomy specimen reveals atypical columnar epithelium that has grown in a tubular manner; **b** (× 10) – Prominent lymphovascular space invasions can be seen over the uterine wall; **c** (× 4) and **d** (× 40) – Vulvar biopsy reveals small atypical cells with necrosis, a high nucleocytoplasmic ratio, and a proliferating solid mass. The equipment and acquisition software used to capture the microscopy images were DP22 standalone-configration system (OLYMPUS, Tokyo, Japan)

An 82-y-old woman, gravida 2 para 2, suffering from right vulvar swelling and pus drainage for 1 week was transferred to our hospital. She presented with a remarkable swelling of her vulva as well as her right leg. Computed tomography scan and magnetic resonance imaging showed a 75 mm irregular mass in her right vulva; additionally, her pelvic and inguinal lymph nodes were markedly swollen. However, we did not detect any instance of distant metastases, and neither did the patient have a family history. Incidentally, the patient had been diagnosed with endometrial cancer stage IA, and she had undergone a total abdominal hysterectomy and bilateral salpingo-oophorectomy 3 y ago. Histological diagnosis of the hysterectomy specimen had confirmed endometrioid carcinoma (grade1, pT1a cN0 cM0) (shown in Fig. 1a). However, lymphovascular space invasion was observed over the uterine wall (shown in Fig. 1b). No adjuvant therapy had been administered post-surgery. The current vulvar biopsy revealed small atypical cells with necrosis, a high nucleo-cytoplasmic ratio, and a proliferating solid mass (shown in Fig. 1c, d). The patient’s tumor marker levels were as follows: cancer antigen 125 – 20.2 U/mL (normal value: ≤ 35.0 U/mL), cancer antigen 19–9 < 2.0 U/mL (normal value: ≤ 37.0 U/mL), squamous cell carcinoma antigen – 1.0 ng/mL (normal value: ≤ 1.5 ng/mL), neuron-specific enolase > 370 mg/mL (normal value: ≤ 16.3 mg/mL), and pro-gastrin releasing peptide – 53.4 pg/mL (normal value: ≤ 80.00 pg/mL). The vulvar SCNEC was thought to be a primary or secondary SCNEC. The tumors grew daily. However, owing to the patient’s age and poor prognosis, only palliative care was provided for the pain management. Unfortunately, the patient died of the disease within 1 month.

### Immunohistochemical and virological analyses

**Fig. 2:**
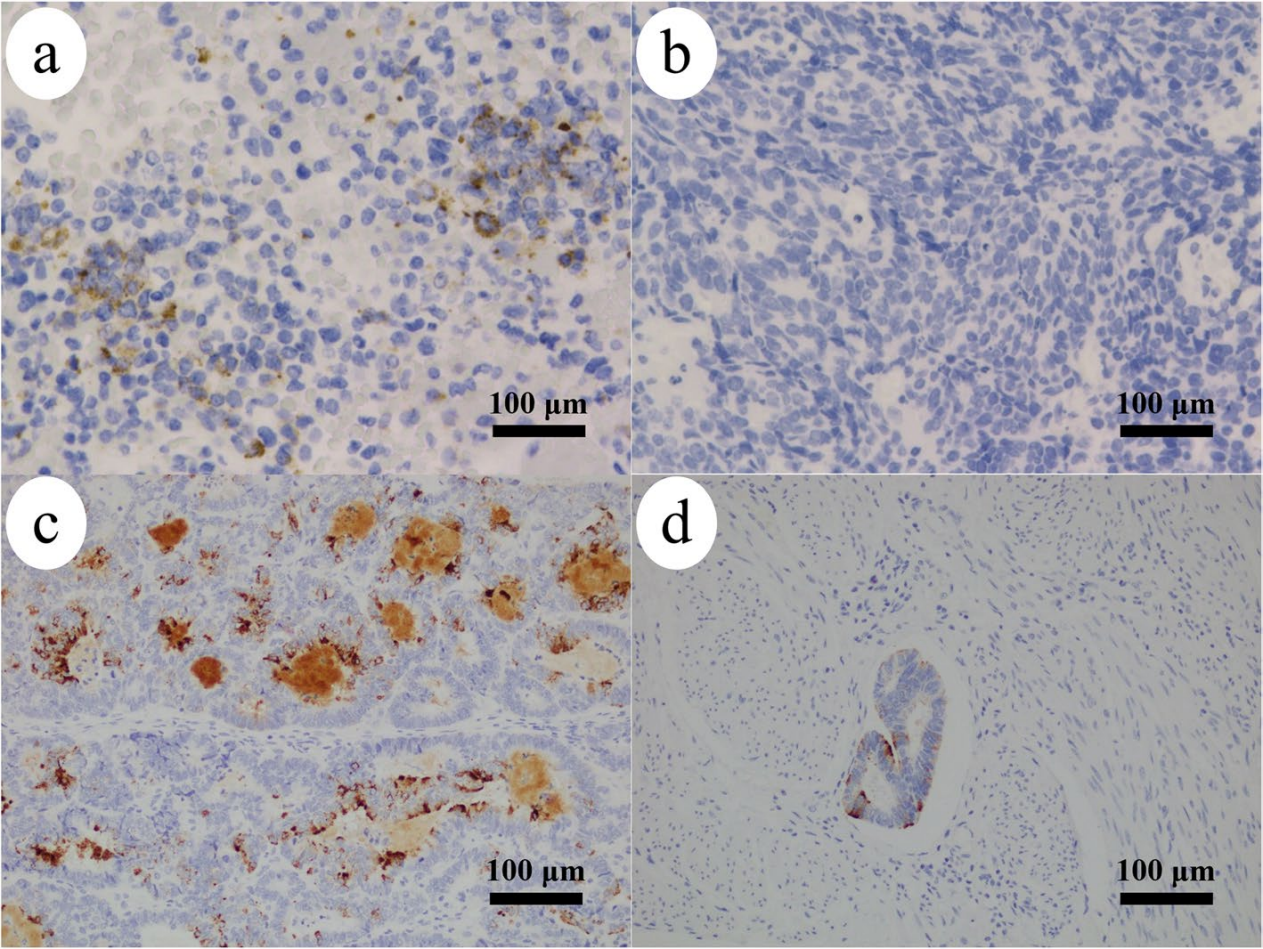
Immunohistochemical findings: **a** (× 40) – Vulvar tumor is positive for chromogranin A; **b** (× 40) – Vulvar tumor is negative for Merkel cell polyomavirus (MCPyV); **c** (× 40) – Endometrial cancer is positive for chromogranin A. **d** (× 40) – Endometrial cancer cells infiltrated into lymphatic vessels are also positive for chromogranin A. The equipment and acquisition software used to capture the microscopy images were DP22 standalone-configration system (OLYMPUS, Tokyo, Japan)

**Fig. 3:**
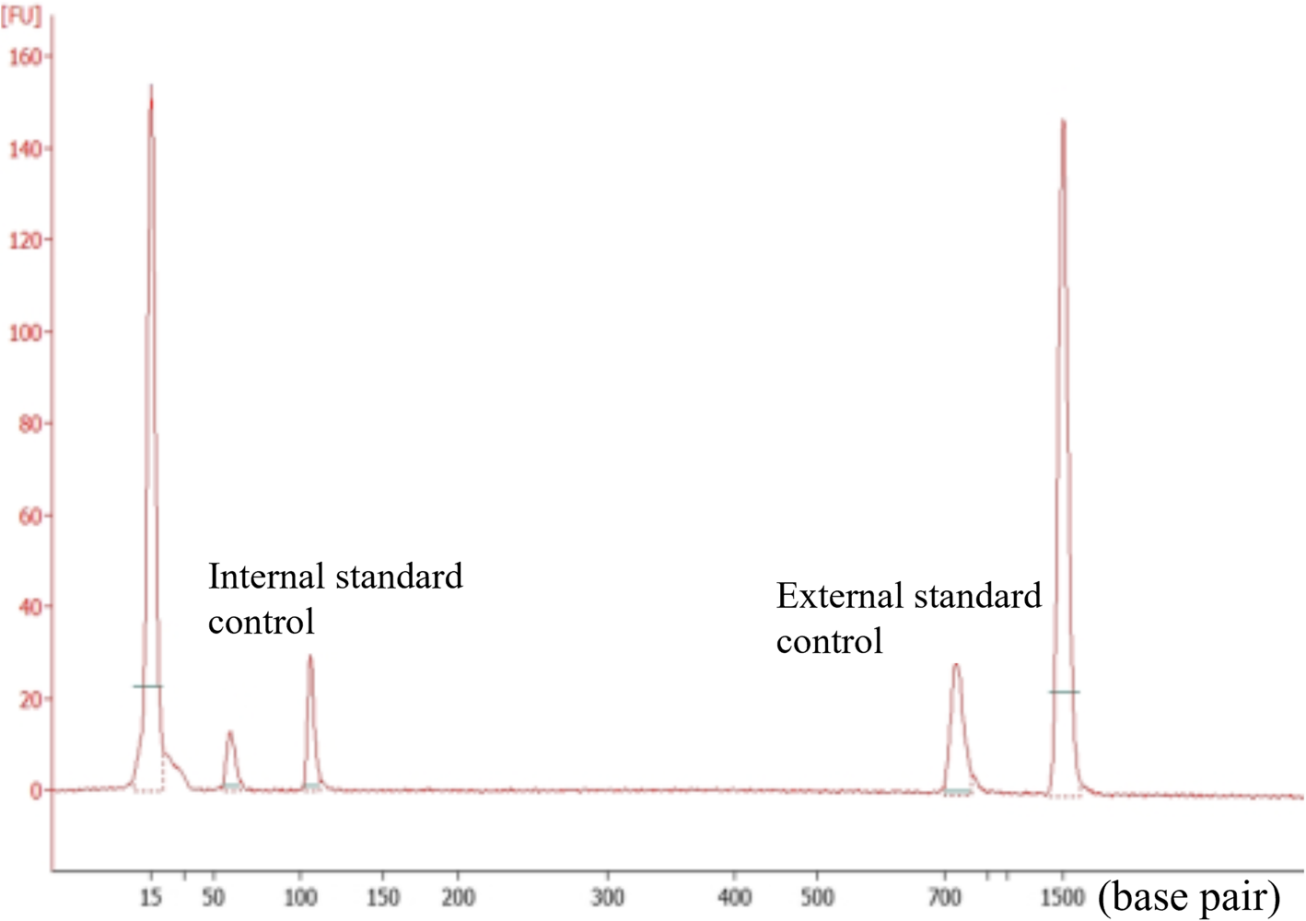
Human papillomavirus (HPV) DNA typing analysis (types 6, 11, 16, 18, 30, 31, 33, 35, 39, 45, 51, 52, 56, 58, 59, and 66) of the vulvar tumor is negative. No amplified gene was found between the internal standard control (approximately 100 base pairs) and external standard control (approximately 700 base pairs)

Immunohistochemical staining of the vulvar mass revealed that most of the vulvar tumor cells were positive for AE1/AE3, CAM5.2, neuron-specific enolase, CD56, and chromogranin A (shown in Fig. 2a). On the contrary, the tumor cells tested negative for MCPyV (shown in Fig. 2b), CK20, synaptophysin, c-kit, estrogen receptor, progesterone receptor, p16, and adrenocorticotropic hormone. Retrospectively, immunohistochemical and virological analyses had been performed for the initial endometrial endometrioid carcinoma. Immunohistochemical staining had revealed that the tumor cells were positive for CD56 and chromogranin A (shown in Fig. 2c). Notably, endometrial cancer cells infiltrated into lymphatic vessels are also positive for chromogranin A. Additionally, the tumor cells were negative for MCPyV and CK20. Furthermore, the HPV DNA typing analysis (types 6, 11, 16, 18, 30, 31, 33, 35, 39, 45, 51, 52, 56, 58, 59, and 66) of the formalin-fixed, paraffin-embedded specimens of the vulvar tumor was negative (PapiPlex™, GeneticLab Co., Hokkaido, Japan) (shown in Fig. 3). Therefore, the vulvar tumor was suggested to be a recurrence of the endometrial cancer rather than a primary vulvar HGNEC (shown in Table 1).

**Table 1:** Comparison between primary vulvar SCNEC/MCC (from reference 1) and the present case

Cases	CK20	McPyV	HR-HPV
SCNEC #1	Negative	Negative	Positive
SCNEC #2	Negative	Negative	Positive
SCNEC #3	Negative	Negative	Positive
SCNEC #4	Negative	Negative	Positive
SCNEC #5	Negative	Negative	Positive
SCNEC #6	Negative	Negative	Positive
MCC #1	Positive	Negative	Negative
MCC #2	Positive	Positive	Negative
MCC #3	Positive	Positive	Negative
MCC #4	Positive	Positive	Negative
MCC #5	Positive	Positive	Negative
MCC #6	Positive	Positive	Negative
The present case	Negative	Negative	Negative

*CK20* cytokeratin 20, *McPyV* Merkel cell polyomavirus, *HPV-HR* high-risk human papillomavirus, *SCNEC* small cell neuroendocrine carcinoma, *MCC* Merkel cell carcinoma

## Discussion and conclusions

It is important to make a differential diagnosis of vulvar HGNECs with small cell morphology with respect to primary Merkel cell carcinomas, primary SCNECs, and secondary/metastatic SCNECs originating from other organs. In the present case, the patient had a history of endometrial endometrioid carcinoma. Notably, the coexistence of endometrial endometrioid carcinoma and HGNEC is a well-known phenomenon [2–4]. Moreover, in case of recurrent metastasis, the endometrial endometrioid carcinoma occasionally presents as a dedifferentiated cancer [5] or HGNEC [3, 4] components at the site of metastasis/recurrence. Actually, Shimazaki I et al. [6] reported that secondary small cell carcinoma in vaginal stump after hysterectomy for endometrial endometrioid carcinoma. Therefore, metastasis/recurrence of endometrial endometrioid carcinoma cannot be ruled out based upon only morphological differences.

Merkel cell carcinoma and SCNEC are often morphologically indistinguishable. Chen et al. [1] reported that immunohistochemical observations (CK20 and MCPyV) as well as tests for HR-HPV infection are useful for differential diagnosis of HGNECs. Primary vulvar Merkel cell carcinoma is positive for CK20 (paranuclear dot-like and/or ring-like reactivity) and MCPyV, but negative for HR-HPV. On the contrary, primary vulvar SCNEC is positive for HR-HPV, but negative for CK20 and MCPyV [1] (shown in Table 1). In the present case, the vulvar HGNEC tested positive for chromogranin A, but it was negative for CK20, MCPyV, and HR-HPV. These immunohistochemical and virological analyses indicated that the vulvar HGNEC in the 82-y-old patient was neither primary vulvar SCNEC nor Merkel cell carcinoma.

In the present case, the patient had been previously detected with an early-stage, low-grade endometrioid carcinoma, and she had presented with prominent lymphovascular space invasion over the uterine wall. Endometrial cancer in the elderly can be aggressive, even in early-stage low-grade [7]. Vulvar metastasis can occur even in early-stage and low-grade endometrioid carcinomas [8], and prominent lymphovascular space invasion is also consistent with metastases to the vulva and lymph nodes. Additionally, the endometrial endometrioid carcinoma was positive for the neuroendocrine marker chromogranin A. Notably, the tumor cells in the lymphovascular space invasion are also positive for chromogranin A. These observations support the phenomenon that the endometrial cancer had metastasis/recurrence to the vulva and lymph nodes. However, this study is based upon a single patient, and it also lacks a detailed molecular analysis of the vulvar mass since only small biopsy specimens could be obtained from the vulvar tumor. Moreover, it did not contribute to the patient's treatment strategy. This patient was unable to undergo surgery or chemotherapy because of her age and general health condition. Incidentally, endometrial HGNECs often exhibit a high microsatellite instability [9] and an abnormal expression of DNA mismatch repair proteins [2]; hence, immune-checkpoint inhibitors via PD-1/PD-L1 may be an effective line of therapy in such cases [10]. Therefore, for the benefit of future patients, it is essential to develop a method of distinguishing between primary and secondary/metastatic vulvar cancers.

In conclusion, we report a vulvar neuroendocrine carcinoma that is independent of both Merkel cell polyomavirus and human papillomavirus, thereby suggesting a recurrence of endometrial cancer. It can be concluded that along with morphology-based studies, immunohistochemical and virological analyses, including CK20, MCPyV, chromogranin A, and HR-HPV, are useful for the differential diagnosis of HGNECs as primary Merkel cell carcinomas, primary SCNECs, and secondary/metastatic SCNECs from other organs.

## Data Availability

All data generated or analyzed during this study are included in this published article.

## Abbreviations

HGNEC: High-grade neuroendocrine carcinoma
SCNEC: Small cell neuroendocrine carcinoma
McPyV: Merkel cell polyomavirus
CK20: Cytokeratin 20
HPV-HR: High-risk human papillomavirus
